# Inhalable Formulations to Treat Non-Small Cell Lung Cancer (NSCLC): Recent Therapies and Developments

**DOI:** 10.3390/pharmaceutics15010139

**Published:** 2022-12-31

**Authors:** Chetna Gupta, Aadya Jaipuria, Nikesh Gupta

**Affiliations:** 1 Department of Chemistry, Hansraj College, University of Delhi, Delhi 110007, India; 2 Department of Pharmaceutical Sciences and Experimental Therapeutics, College of Pharmacy, University of Iowa, Iowa City, IA 52242, USA; 3 Massachusetts College of Pharmacy and Health Sciences, Boston, MA 02115, USA

**Keywords:** inhalation, pulmonary delivery, lung cancer, dry-powder inhalers, nanomedicines

## Abstract

Cancer has been the leading cause of mortalities, with lung cancer contributing 18% to overall deaths. Non-small cell lung cancer (NSCLC) accounts for about 85% of all lung cancers. The primary form of therapy used to treat lung cancer still includes oral and systemic administration of drugs, radiotherapy, or chemotherapy. Some patients have to go through a regime of combination therapy. Despite being the only available form of therapy, their use is limited due to the adverse effects, toxicity, and development of resistance over prolonged use. This led to a shift and progressive evolution into using pulmonary drug delivery systems. Being a non-invasive method of drug-administration and allowing localized delivery of drugs to cancer cells, inhalable drug delivery systems can lead to lower dosing and fewer systemic toxicities over other conventional routes. In this way, we can increase the actual local concentration of the drug in lungs, which will ultimately lead to better antitumor therapy. Nano-based systems also provide additional diagnostic advantages during lung cancer treatment, including imaging, screening, and tracking. Regardless of the advantages, pulmonary delivery is still in the early stages of development and various factors such as pharmacology, immunology, and toxicology should be taken into consideration for the development of suitable inhalable nano-based chemotherapeutic drugs. They face numerous physiological barriers such as lung retention and efficacy, and could also lead to toxicity due to prolonged exposure. Nano-carriers with a sustained drug release mechanism could help in overcoming these challenges. This review article will focus on the various inhalable formulations for targeted drug delivery, including nano-based delivery systems such as lipids, liposome, polymeric and inorganic nanocarriers, micelles, microparticles and nanoaggregates for lung cancer treatment. Various devices used in pulmonary drug delivery loaded on various nano-carriers are also discussed in detail.

## 1. Introduction

Cancer is a leading cause of morbidity and mortality worldwide. According to the American Cancer Society, in the United States, 1.9 million new cases of cancer and 0.6 million cancer deaths are estimated in 2022 [1]. Lung cancer is in the lead in terms of both mortality and incidence, causing approximately 350 deaths per day in the United States [1]. Lung cancer has a higher mortality rate than breast, prostate, and pancreatic cancer. According to Global Cancer Observatory (GLOBOCAN) estimates for 2020, lung cancer accounts for 11.4% of all reported cancer cases and accounts for 18% of all cancer-related deaths. Furthermore, the estimated number of deaths from 2020 to 2040 worldwide are predicted to increase by 63.8% in both sexes combined, rising by 64.8% in males and 61.9% in females.

Lung cancer develops and spreads due to genetic and certain microenvironmental factors. Tobacco consumption, however, remains the leading risk factor for lung cancer, accounting to majority of deaths in the United States. Tobacco smokers have a tenfold increased risk of developing lung cancer compared to nonsmokers [2]. Exposure to radon gas accumulated in the air after being released from soil is the second leading cause of lung cancer. Exposure to second-hand smoke, industrial hazard metals (such as arsenic, chromium, cadmium), some organic toxins, and environmental hazards such as radiation, poor air quality, diesel exhaust, and asbestos are also factors responsible for this disease. A history of respiratory illnesses and occupational exposures (rubber manufacturing, painting, and chimney sweeping) makes one more susceptible to developing lung cancer.

The classification of lung cancer on the basis of histology can be made into small-cell lung carcinoma (SCLC) and non-small-cell lung carcinoma (NSCLC) established on the types of cells observed using a microscope. NSCLC accounts for 85% among all cases of lung cancer with SCLC contributing the remainder 15%. Non-small cell lung cancer is further subdivided into squamous cell carcinoma, adenocarcinoma, and large cell carcinoma based on histology. Adenocarcinoma accounts for approximately 40% of all non-small lung cancer cases and is most commonly observed in nonsmokers [3].

The current conventional therapies available for lung cancer are failing because they are only effective in the preliminary stages of the disease. However, the onset of symptoms often happens in the final stages of lung cancer, and over 70% of patients are diagnosed well after the spread of the tumor from the primary site (the lungs), making treatment difficult [4]. The stage of lung cancer, as well as the patient’s physical condition, play an important part while choosing a treatment plan [5]. Currently, the treatment options on hand are surgery, chemotherapy, radiation therapy, immunotherapy, and laser therapy. Only a minority of patients are eligible for surgical resection as a viable treatment plan. This is because 55% of all patients have metastatic (state IV) cancer when a diagnosis is provided. Chemotherapy is recognized as the most integral part and forms the foundation of treatment in such cases. Platinum-based therapy is the backbone of chemotherapy for NSCLC and is administered in amalgamation with chemotherapeutic drugs (paclitaxel, vinorelbine, topotecan, gemcitabine) [3]. Conventional therapy includes intravenous administration of chemotherapeutic drugs. This has limited flexibilities because of the adverse aftereffects associated with it and emergence of resistance over time [6,7]. Chemotherapy often leads to nausea and vomiting, which are the most feared side effects, as well as gastrointestinal side effects, anemia, neuropathy, and neurotoxicity [7]. Intravenous administration results in exposure of the entire body to toxic agents and low drug concentration at tumor sites. Hence, there is an emerging need to investigate better treatment plans for curing lung cancer and improving quality of life. Targeted drug delivery directly to the lungs in order to decrease systemic circulation of the drugs, improve healthy cell viability, and increase toxicity of tumor cells is the need of the hour.

Pulmonary drug delivery via inhalable formulations is a noninvasive and targeted route of administration suitable for lung cancer therapy. Aerosols, dry-powder inhalers (DPIs), and liposomes can also be formulated with ideal physicochemical properties to reach lung tumors via different pathways and target deep sites in the lungs. Nanotechnology has a broad horizon and potential to be used in cancer therapy [8]. Nano-carriers can be developed to entrap the drugs and carry out intratracheal applications to specifically target the cancerous cells in the lungs.

In this paper, we will discuss the various advantages, challenges, and toxicity encountered in the application of inhalable formulations for the treatment of lung cancer. The various physicochemical properties that must be taken into consideration for development of these formulations have also been touched upon. The different carriers for these formulations have been trifurcated on the basis of their size into nanocarriers, microparticles, and nanoaggregates and nanocomposites with the main focus on the former. The nanoparticles have further been divided into categories based upon their shapes, size, and lipophilicity.

## 2. Advantages and Challenges of Inhalable Anticancer Therapy

Having pulmonary delivery routes and using inhalable non-invasive formulations have various advantages. It is a viable treatment plan for patients with surgically unresectable tumors due to location or stage of cancer, can be self-administered, and is non-invasive. Conventional routes of administration (oral or intravenous) cause a greater percentage of hepatic metabolism due to a high concentration of enzymes in the liver and gastrointestinal tract, and hence a lower bioavailability. Lungs possess a limited number of enzymes that partake in metabolism, decreasing metabolism significantly and hence increasing bioavailability of the drug. Since the drug is not required to circulate systemically, there is less toxic exposure to healthy tissues thereby decreasing cytotoxicity [9]. Since the drug reaches the tumor site directly, there is a rapid onset of action. In addition, there is an increased absorption of drug from the lungs owing to the broad surface are of the alveoli and thin width. All this improves absorption, leads to a faster onset of action, and requires lower doses [10,11].

When loaded on lipid-based carriers, these drugs can directly penetrate the tumors irrespective of their site in the lung. The inhaled drug can reach squamous and bronchioloalveolar cell cancer types directly since they are located near the airway [12]. For smaller lung tumors located away from the main airways, the drugs can penetrate the local blood supply and reach them via the interlink between the bronchial and pulmonary circulations [13].

Regardless of all the advantages over conventional therapy, pulmonary delivery has certain drawbacks that need attention. One major risk involved is pulmonary toxicity due to the high concentration of drugs in the lungs [14]. Lung cancer patients usually have a pre-existing lung condition due to tobacco use and hence have weaker lungs. This affects their ability to tolerate various drugs and are at high risk of developing chemotherapy-induced lung diseases [7,15,16].

**Figure 1:**
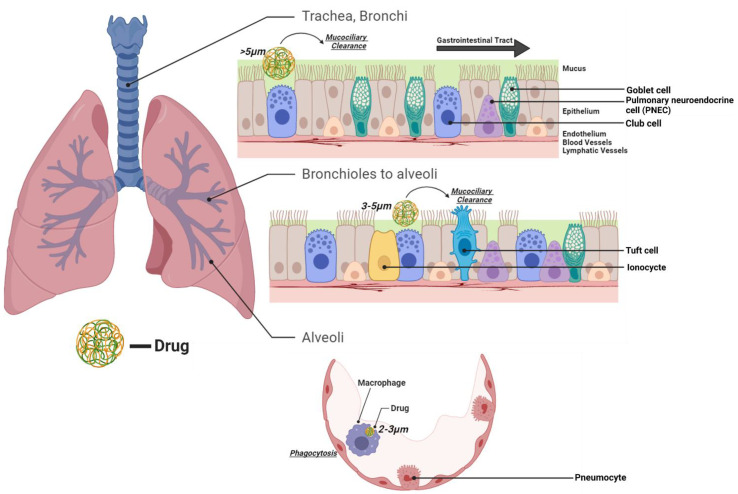
The clearance mechanisms involved at different sites in the lungs.

Lung clearance mechanisms (Figure 1), although beneficial in protecting the lungs from xenobiotic substances, present a possibility of removing the drug from the lungs prior to it having an effective cytotoxic action on the tumor cells. Mucociliary clearance is our body’s primary mechanism to clear any foreign substances from the pulmonary tract. The particles are carried upwards through the bronchus to the larynx and into the gastrointestinal tract. Of all inhaled particles, 80–90% are cleared within 24 h of administration via the mucociliary clearance mechanism [17]. The mucous layer becomes thicker in patients with a pre-existing lung condition increasing mucociliary clearance, making the drugs even less effective. Some physicochemical properties of the drug, such as a high partition coefficient (log P), suggesting high lipophilicity, may cause the drug to become entrapped in the mucous and eliminated from the lungs instead of reaching the tumor. Particles of size 1.5–3.0 μm are cleared from the respiratory zone by alveolar macrophages via phagocytosis, or “cell eating”, but it is inefficient to clear nanoparticles (<200 nm). Lastly, the drugs may be dissolved, upon which they can either become enzymatically degraded or find their way into systemic circulation [18,19]. There are few suggested formulation strategies that can be applied to overcome the removal of the drug(s) via clearance mechanism (Table 1). Hereby, it is crucial to analyze the physicochemical properties of the drugs in order to enhance therapeutic effects and minimize clearance by either of the mechanisms.

## 3. Physicochemical Properties

Physicochemical properties, as discussed previously, are extremely important in determining the duration of action, bioavailability, and ultimately the therapeutic efficiency of a drug.

### 3.1. Size of Nanoparticles

The efficacy of drugs depends heavily on the size of the nanoparticles. Having an aerodynamic diameter (D_ae_) > 5 μm results in the particle being trapped in the upper respiratory tract (bronchi, trachea) and cleared via the mucociliary mechanism. Particles sized < 0.5 μm can diffuse into the bloodstream and get eliminated via the respiratory airflow. A particle of size in the order of 200 nm is often cleared through phagocytosis by alveolar macrophages and hence nanoparticles sized between 100–150 nm is ideal for pulmonary delivery [25]. The enhanced permeability and retention (EPR) effect of particles with size in the order of sub-microns also leads to an increased drug concentration at the tumor site since it leaks out through the blood and accumulates in the tumors [25].

### 3.2. Surface Properties

Particles with a large surface area, i.e., bigger in diameter, can have high contact surface with cancer cells, resulting in higher bio-interactions. The cell membranes of tumor cells are often negatively charged and attracted towards positively charged surfaces. Hence, cationic nanoparticles have a higher binding tendency with the tumor cells [26]. Anionic and neutral particles are taken up rapidly by the lymph nodes, hence radically lessening the time they spend at the tumor site. Cationic nanoparticles have a greater propensity to be taken up by the epithelial cells or macrophages in the lungs and tend to be retained longer within the lungs [27].

### 3.3. Lipophilicity

A drug needs to have a longer lung residence time in order to have a therapeutic effect. A particle with a log P > 0 is more lipophilic, therefore has a higher efficacy for the lipid membrane present in the lungs and is envisaged to be rapidly absorbed. This limits the drug exposure to the tumor; therefore, it is vital that the drug be hydrophilic with a log P < 0 [28]. This can be achieved by modifying formulations and by using salt formulations of drugs [29].

### 3.4. Nanoparticle Surface Modifications

The surface of nanocarriers can be modified with various ligands and functional groups. Proteins, peptides, or antibodies can be added onto nanoparticles to improve specific binding with cancer cells via receptor proteins. In this way an internalization, and localization of the particles to the tumors can be achieved, and a lower dose formulation can be made [28].

## 4. Inhalable Formulations

A variety of inhalable formulations have been developed; regrettably, not all are appropriate for intratracheal administration of drugs. A few of these delivery systems include microparticles, nanoparticle carriers, nanoaggregates, and nanocomposites.

### 4.1. Nanocarriers

Nanocarriers have shown great promise in terms of being developed into successful drug delivery systems. This is owing to their ability to target cancer tissues directly in the lungs and help in sustained drug release. Nanoparticles can also avoid mucociliary clearance and alveolar macrophage phagocytosis, thereby significantly increasing lung residence time of the drugs. These nanocarriers are of the same size as that of biological components and hold the capacity to target them at the surface or at their core by penetrating through their membranes.

#### 4.1.1. Polymeric Nanoparticles

Polymers of various compositions make up a massive portion of nanotherapeutics used for chemotherapeutic delivery via the pulmonary route. They are especially advantageous in escaping alveolar macrophage uptake and hence can reside in the lungs for longer. Drugs are physically entrapped into inhalable, biodegradable nano polymers, which are then administered through the pulmonary route [30]. Some polymeric nanoparticle systems employed for inhalation delivery include polylactic-co-glycolic (PLGA)- and polyethylene glycol (PEG)-based aerosol dry powder formulations [31]. Coating drug particles with PEG creates a hydrophilic barrier around the particle and increases mucus penetration while decreasing mucoadhesion. This helps in the prevention of clearance of drugs by the mucociliary mechanism and also to some extent by lung macrophages [32].

Wang et al. loaded a resveratrol–cyclodextrin complex onto PLGA polymers (CD-RES NP) in order to improve hydrophilicity and chemical and physical stability. These particles were also successfully aerosolized with an aerodynamic diameter (D_ae_) of 220 nm. The TEM imaging showed that CD-RES NPs had a spherical shape. It was concluded that by doing so, the therapeutic efficiency of resveratrol was improved while retaining its antioxidant activity. A heightened cellular uptake, cytotoxicity, and increased anticancer activity were also observed [33].

Sorafenib (SF)-loaded inhalable PLGA nanoparticles were developed by Shukla et al. Their aim was to optimize SF for localized treatment against NSCLC [34]. The emulsion had a single oil/water base and was prepared by the solvent evaporation technique, and they coated the nanoparticle with a cationic poly-L-arginine (PLA) polymer. The coated particles were found to be 192.6 ± 5.0 nm in size and have a spherical morphology. Because of the electrostatic interactions between the cation polymer and anionic phospholipid bilayer of tumor cells, the coated sorafenib nanoparticle (SF NP) demonstrated enhanced cellular uptake. These SF NPs demonstrated superior therapeutic efficacy at low doses as well as an appropriate aerosolization efficiency for pulmonary delivery. The researchers also discovered that SF NPs substantially inhibited NSCLC cell metastasis [34].

A pH-triggered release system for doxorubicin was designed using polymeric nanocarriers. The nanoparticle used was composed of alginate/chitosan, which was then PEGylated. Doxorubicin was linked to this via an acid–labile amide bond. For every mg of nanoparticle, 444.3 ± 9.2 µg doxorubicin was loaded onto it. This system had a hydrodynamic diameter of 205.7 ± 15.0 nm, and its zeta potential was found to be −25.17 ± 2.67 mV. The SEM images showed that the morphology of the formed particles was spherical. These properties indicated the possibility of using the DOX-PEG-NPs for intratracheal administration in the dry powder form. Upon carrying out drug-release studies at pH 5.5 and 7.4 media, 10% and 6.3% drug was released in the first 5 h, respectively. Drug release was faster in acidic medium but still slower than that of free doxorubicin (70–85% in the first 5 h). An MTT assay performed on A549 lung cancer cells showed significant toxicity of DOX-PEG-NPs in a dose dependent manner. Therefore, these polymeric carriers are promising candidates to be developed into a successful formulation to treat NSCLC [35].

Amodiaquine (AQ), an antimalarial drug was repurposed and inhalable nanoparticulate formulation was developed to explore its activity against NSCLC. A high-pressure homogenization (HPH) method was used to create biodegradable PLGA-based NPs. They were then coated with a cationic polymer—polyethylenimine (0.5% *w*/*v*). The nanoparticles formulated hence were in liquid state and had an aerodynamic diameter of 4.7 ± 0.1 μm. Cytotoxicity studies conducted on A549 cells showed that the NPs had a lower IC_50_ as compared to the free drug. The study revealed that amodiaquine can be repurposed to treat NSCLC [36].

Another approach followed is the covalent coupling of the drug onto a biological polymeric carrier. Bovine serum albumin (BSA) is the most common biopolymer utilized for this purpose. Due to the specific binding of albumin to its receptors, albondin and SPARC (secreted protein, acidic and rich in cysteine), these formulations could be directly targeted to the tumor tissues [30]. Quinacrine has recently been found to possess anticancer properties, however, it shows low bioavailability and permeability at the tumor site and leads to side effects such as skin pigmentation and rashes. To overcome these drawbacks, Vaidya et al. synthesized quinacrine-loaded cationic PLGA nanoparticles. The method employed for this was an emulsion solvent evaporation method. Stabilization was carried out with 2% polyethyleneimine (PEI). Prepared nanoparticles were cationic and had a size of 224 ± 14 nm. These cationic PLGA nanoparticles were then coated with BSA. This was carried out to control the positive charge and increase receptor-mediated uptake of nanoparticles by the tumor cells. This also led to a slower drug release than in the case of uncoated PLGA nanoparticles. The BSA-coated PLGA nanoparticles had a spherical morphology and when aerosolized had a D_ae_ of 4.11 ± 0.26 μm, displaying good aerosolization potential. A 1.5-fold higher cellular uptake by A549 cells was observed for BSA-coated nanoparticles compared to plain NPs, which were significantly toxic. The former also showed negligible toxicity on human embryonic kidney cells (HEK293), thereby making BSA-coated PLGA quinacrine a potential chemotherapeutic agent for the treatment of NSCLC [37].

Sodium alginate is a sodium salt of alginic acid, making it highly water-soluble, capable of cross-linking with bivalent cations, such as Ca^2+^, resulting in an insoluble network [38,39]. Calcium alginate microparticles are an appealing system for the development of drug carriers and controlled release drug delivery systems. This is due to their biodegradability and biocompatibility, low toxicity, high availability, and affordability. In addition, calcium alginate is mucoadhesive, hence can be employed for sustained and prolonged site-specific drug delivery of drugs to mucosal tissues [38,39,40,41,42].

Alipour et al. fabricated alginate microparticles using an emulsification/gelation method and characterized it using a Shimadzu particle size analyzer and SEM imaging [43]. The microparticles hence formed had a mean volume diameter of 3 ± 0.7 μm, mass median aerodynamic diameter of 5.9 ± 0.33 μm, fine particle fraction of 13.9 ± 0.57%, and encapsulation efficiency of 61 ± 4%. An MTT assay was used to evaluate in vitro cytotoxicity towards A549 cells. It was observed that paclitaxel loaded microparticles had similar activity as that of free paclitaxel [44].

Chitosan is a cationic polysaccharide derived from chitin [43]. It is a biopolymer and its application in intratracheal delivery of chemotherapeutics is widespread due to its biocompatibility and biodegradability. Chitosan also possesses antimicrobial properties [45]. Singh et al. formulated chitosan microspheres from an emulsion and loaded it with cisplatin using an ionotropic gelation method [46]. These drug-loaded chitosan–cisplatin microspheres had a mean particle size of 5.2 ± 1.19 µm, aerodynamic diameter D_aero_ of 2.71 µm with ~79.2 ± 2.9% drug content, and a fine particle fraction (FPF) of 33.4 ± 2.4%. In vitro cytotoxicity studies were performed on A549 cells. The microspheres showed higher IC_50_ values as compared to free cisplatin since the former leads to a slow drug release. Hence, cisplatin-loaded chitosan microspheres can be optimized further to be used as an inhalable formulation [46].

Rosiere et al. synthesized a folate-grafted copolymer consisting of chitosan and polyethylene glycol (PEG) and coated it with a previously developed paclitaxel-loaded solid lipid nanoparticle (SLN) [47]. The hence produced formulation (F-PEG-HTCC-coated SLN) had a 100% encapsulation efficiency, a mean diameter of 250 nm, and a zeta potential of +32 mV. It was observed that the paclitaxel from the coated SLN was released in a sustainable form and its residence in the lung was prolonged up to 6 h. F-PEG-HTCC-coated SLN also significantly reduced the IC_50_ value of paclitaxel in M109-HiFR cells. Therefore, the produced formulation was advantageous for pulmonary delivery of paclitaxel [47].

Fucoidan is a polysaccharide with L-fucose monomers and is sourced from extracellular matrix of brown seaweed (Phaeophyta) and is also found in sea urchins and sea cucumbers [48]. The use of fucoidan is widespread in drug formulation due to its antioxidant, anti-inflammatory, and antithrombotic properties. They were also found to be non-toxic when orally administered at a dose of 300 mg/kg per day for 6 months in sub-chronic toxicity studies [49]. In lung cancer, fucoidan is known to reduce tumor cell proliferation. Hsu et al. carried out in vitro and in vivo studies using lung cancer cells and found that fucoidan reduces transforming growth factor receptor (TGFR) protein levels. It also inhibited the in vitro growth of lung cancer cells when administered orally in LLC1-bearing mice. Hence, fucoidan can be used as a potential dietary supplement and therapeutic agent for treatment of lung cancer [50].

#### 4.1.2. Dendrimers

**Figure 2:**
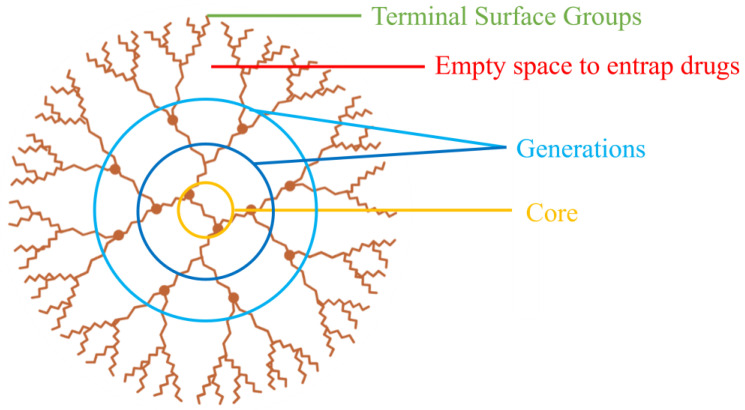
Structure of dendrimers.

The name dendrimer is derived from a Greek word “Dendron” meaning “tree”. These particles are named so owing to their three-dimensional structure, which forms a tree structure of hyperbranched homopolymers (Figure 2) [51]. Dendrimers have four main areas: (i) a main core, (ii) interior layers called generations that consist of a repeating unit attached to the core, (iii) terminal surface groups, and (iv) spaces in which the drug may be entrapped. They resemble micelles in a way that they are constituted of an apolar core and a polar shell, hence are often called “unimolecular micelles” [9].

Dendrimeric drug carriers are often sized in the range of 4 to 20 nm [52]. This affords dendrimers an upper hand over polymeric nanocarriers, since they have better probability of tumor penetration, more efficient interstitial diffusion, and extent of absorption. Dendrimers can conjugate with drugs either by (i) encapsulation of the drug into their dendrimeric cavities, or (ii) by surface binding between the two. The interactions holding the dendrimer and drug together can be electrostatic interactions, hydrophobic, or hydrogen-bonding in case of entrapment and electrostatic or covalent bonding in case of surface conjugation [53].

The dendrimer surface can be also functionalized by attaching functional groups such as antibodies and ligands. This increases the specificity of dendrimers to lung tumors via receptor-based binding [54]. Dendrimers can also be loaded onto polymers to make them more biodegradable and selective [52]. Low molecular weight dendrimers (<20 kDa) can be degraded into in the lungs and be absorbed faster after pulmonary delivery. In contrast, large molecular weight PEGylated dendrimers improve metabolic stability and increase retention time in the lungs [52]. As a result, PEGylated dendrimer-based carriers can be used for targeted and controlled delivery to tumor sites inside the lungs. This was demonstrated by Kaminskas et al., who synthesized a 56 kDa biodegradable PEGylated polylysine dendrimer and conjugated it with doxorubicin. This was carried out in order to increase the lung residence time of doxorubicin and promote its controlled release. When rats were intratracheally administered dendrimer–drug (D-DOX) formulation, there was rapid removal of the formulation from the lungs, approximately 60% within the first 24 h. The drug was cleared by a combination of mucociliary clearance out of the lungs and absorption of the intact formulation into the blood stream from the lungs. After 24 h, biodegradation of the dendrimer was observed, as well as clearance due to absorption. Clearance is an important factor to consider when designing formulations, since it minimizes lung toxicity and prevents accumulation of the drug over long periods of time. To test the anticancer activity of inhaled D-DOX against lung resident cancer, the MAT 13762 IIIB rat model of lung metastatic breast cancer was used. D-DOX was administered for two weeks, twice a week, and a 95% reduction in lung tumor was observed as compared to only a 30–50% reduction when doxorubicin was administered intravenously. Plain doxorubicin also caused extensive lung-related toxicity and death within several days of a single dose. Hence, PEGylated dendrimeric formulations have potential to be administered via the pulmonary route and can be used for prolonged exposure of tumor to chemotherapeutic drugs and to improve anticancer activity [52].

#### 4.1.3. Lipid-Based Nanocarriers

The amount of research being carried out to develop lipid-based nanocarriers for pulmonary delivery of chemotherapeutic drugs has been increasing, as they have various advantages over the other carriers. Lipid-based carriers are biodegradable, non-toxic, allow sustained release of drugs, can cross the phospholipid membrane barriers of tumor cells, and can transport across blood vessels [55].

**Figure 3:**
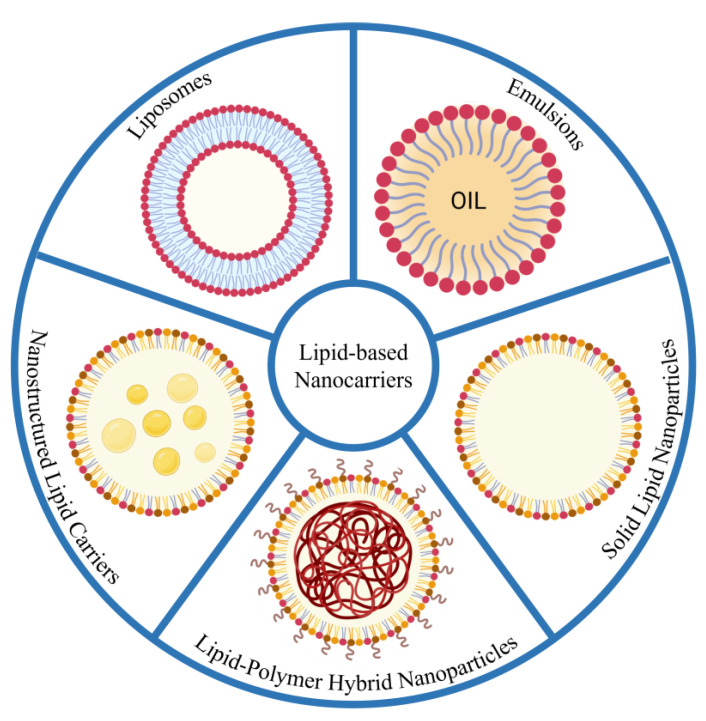
The different lipid based nanocarriers used in pulmonary delivery of chemotherapeutics.

Specific carriers are required to target different tumors in different locations in the lungs. Lipid-based nanocarriers are advantageous since a variety of formulations can be made by tuning their surface properties. Additionally, for drugs with a high log P value, lipid-based carriers are lipophilic enough to facilitate successful encapsulation of the drug [56]. In contrast, majority of newly discovered drugs have unfavorable physicochemical properties, i.e., they are too hydrophilic to cross membranes and often have low oral bioavailability. Encapsulating these in a lipid-based formulation can help alleviate these issues and improve therapeutic efficacy. Each lipid-based carrier has a layer of either phospholipids, a phospholipid bilayer, or a surfactant layer. The distinct structures can be seen in Figure 3.

The use of lipid-based nanocarriers as delivery systems for drugs to treat non-small cell lung cancer has been tested out in several studies and promising results have been obtained (Table 2). The importance is placed specifically on liposomes, solid lipid nanoparticles (SLNs), nanostructured lipid carriers (NLCs), and lipid–polymer hybrid nanoparticles (LPHN). SLNs were developed as alternatives to polymeric nanoparticles and liposomes, while NLCs are known as second-generation lipid carriers developed in order to overcome the drawbacks of first-generation carriers such as SLNs [57].

##### Liposomes

Liposomes are the pioneers of lipid-based carriers designed to date. They consist of a lipid bilayer across an aqueous core and self-assemble into a spherical vesicle. The properties of liposomes can be varied by changing its size, surface charge, the lipid composition, or the preparation method [58]. The word liposome has Greek origins, being derived from the words “lipos” meaning fat and “soma” meaning structure, signifying liposome means a structure of fats with the feasibility of encapsulation [59]. Liposomes can be prepared either from phospholipid bilayers (phosphatidylethanolamine, phosphatidylglycerol, phosphatidylcholine, phosphatidylserine, and phosphatidylinositol) or by using cholesterol, nontoxic surfactants, glycolipids, and long-chain fatty acids. Liposomes are typically made with lipids derived from the yolk of eggs or soyabean oil [60]. Liposomes consist of phospholipid bilayers that mimic human biological membranes and thus exhibit a degree of biocompatibility, biodegradability, and aid in the diffusion of drugs across the plasma membrane. They also have the ability to increase drug concentrations in tumor cells while decreasing drug concentrations in healthy tissues [61]. Liposomes suitable for drug delivery via the pulmonary route are typically 50–500 nm in size [62]. Different liposomes with varying compositions can be prepared, making its application versatile, and their surface is highly tunable and can be functionalized with polymers (mainly poly(ethylene glycol), PEG) to increase the drug delivery efficiency, i.e., they are PEGylated [63].

**Figure 4:**
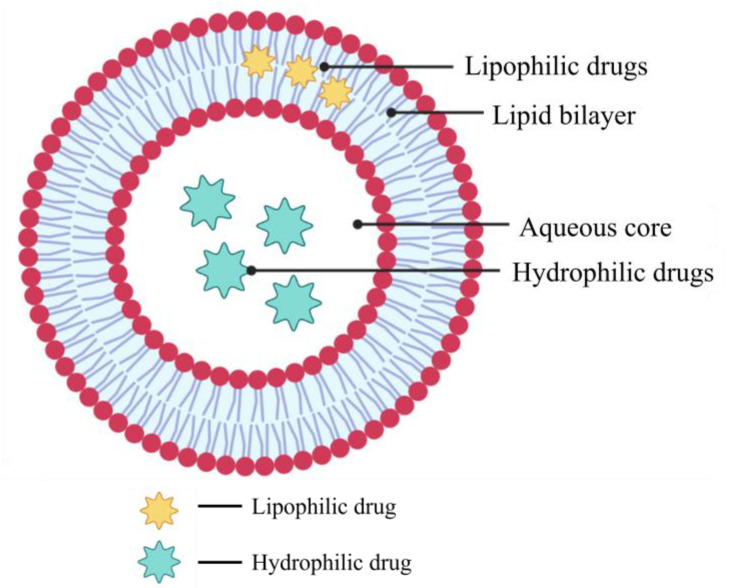
The structure of a liposome encapsulating drugs.

Additionally, because of their unique structure, liposomes have the ability to encapsulate chemotherapeutic agents of varying hydrophilicities; lipophilic drugs can be embedded into the lipophilic bilayer, while hydrophilic drugs can be incorporated into the aqueous core (Figure 4). Overall, liposomes are efficient carriers of anticancer agents—chemotherapeutics, genes, and peptides—and are a promising area of research for pulmonary drug delivery [64].

The delivery of liposomal formulations can be achieved by aerosolization via nebulizers or as a dry powder through dry powder inhalers [65]. Nebulization, however, leads to structural destruction of the liposomes, defeating the purpose. Therefore, dry powder formulations that are stable can be created either by spray-drying, spray-freeze-drying and freeze-drying followed by micronization [66,67].

Zhang et al. created a liposomal curcumin dry powder inhaler (LCD) as a therapeutic agent for primary lung cancer treatment [68]. Curcumin is known to be a potential anticancer agent; however, its use is restricted due to its poor hydrophilicity, poor bioavailability, and rapid clearance from the body. They used a film method to prepare the liposomes, which were composed of soyabean lecithin and cholesterol. Finally, to obtain the LCDs, mannitol was mixed with the liposomes in a lyophilizer and freeze-dried. The LCDs thus obtained were of the order 94.65 ± 22.01 nm and appeared as homogeneous spherical vesicles. The LCDs were also found to be more suitable for pulmonary inhalation than curcumin powder due to their high lung deposition, fine particle fraction of 46.71 ± 5.23%, and an aerodynamic diameter of 5.81 ± 0.06 μm as compared to 13.32 ± 0.36 μm of curcumin powder. Curcumin, already more viable than gemcitabine, when formulated into liposomes shows an even better and faster cellular uptake by human A549 lung cancer cells than free curcumin [69]. Moreover, curcumin-loaded liposomes had a larger selection index than free curcumin as they were highly cytotoxic on A549 cells and did not show any toxicity against healthy human bronchial BEAS-2B epithelial cells. The in vivo studies were carried out on male Sprague–Dawley (SD) rats with lung cancer. The lungs of these rats were sprayed with (a) curcumin liposomal powder, (b) curcumin powder, and (c) gemcitabine through the trachea. The liposomal formulation showed better properties in terms of pathology and the expression of various cancer-related markers such as VEGF, malondialdehyde, TNF-α, caspase-3, and BCL-2 than free curcumin or gemcitabine and hence could be developed further and optimized for intratracheal administration for NSCLC treatment [68].

Pirfenidone (PFD) is an antifibrotic drug that was approved in 2014 for idiopathic pulmonary fibrosis. It was repurposed for treatment of NSCLC by synthesizing a cationic liposomal formulation [69]. The formulations were made using a thin layer hydration technique followed by passive loading of PFD. The resulting formulation, PFD-D-Lip had a particle size of 211.8 ± 12 nm. The TEM images revealed a spherical morphology of the formulation with a uniform size distribution. The prepared formulation had a D_ae_ = 2.59 ± 0.04 µm and an FPF, also called the respirable fraction, of 76.88 ± 2.26, suggesting good aerosolization performance. MTT assay revealed PFD–D-Lip was considerably more cytotoxic towards A549 cells than plain PFD and required in lower doses. In vitro cytotoxicity studies performed on HEK-293 (human embryonic kidney cells) showed no toxicity of PFD-D-Lip formulation. Hence, PFD-loaded liposomes have good potential to be used as a treatment option [69].

Vincristine was co-loaded onto PEGylated liposomal doxorubicin, and its efficacy was studied against NSCLC [70]. The combinatorial liposome was prepared by actively co-loading both vincristine and doxorubicin against a modified ammonium ion gradient. The resulting formulation showed a 95% drug encapsulation of both drugs. The liposomes had a spherical unilamellar structure and had a size of ~94 nm. The dual drug formulation showed better cellular uptake as against liposomal doxorubicin. The cell viability of A549 cells was significantly reduced, and an increased tumor regression was observed compared to in the mono drug liposomal formulation of doxorubicin. As a result, co-encapsulating vincristine into an already developed PEGylated liposomal doxorubicin significantly improved both in vitro and in vivo treatment response against NSCLC [70].

Paclitaxel is an antineoplastic drug that can be used as a possible treatment option for lung cancer patients. PTX is highly lipophilic, making it difficult to solubilize in an aqueous medium for intravenous formulations. Liposomes are a viable alternative to improve pharmacological properties and decrease toxicity. Paclitaxel liposome was prepared in butanol consisting of dilauroylphosphatidyl choline (DLPC) at a drug-to-lipid ratio of 1:10 (*w*/*w*) and lyophilized at −70 °C to prepare DPI formulation [71]. The aerosolized particles were characterized using an Andersen cascade impactor. It was found that they had a mass median diameter of 2.2 ± 0.2 μm. Pharmacokinetic studies showed that the AUC (area under the curve) for the aerosol administration was 26-fold higher than that for intravenous administration via the tail vein. BALB/c mice were inoculated with tumor cells and divided into three random groups. Of the three groups, one was left untreated, one was treated with blank DLPC liposomes, and the last group was administered the PTX formulation intratracheally. The third group of mice treated with liposomal PTX had a lower lung weight than the other groups, suggesting there was significant tumor reduction upon treatment with aerosolized paclitaxel liposomes. Inhalation of the PTX liposomal formulation also led to prolonged survival in the infected mice. All this data suggests good treatment potential of PTX-DLPC for NSCLC [71].

Xiao et al. developed a system for combined chemo–sonodynamic (Chemo-SDT) therapy for the treatment of metastatic LC. They used cationic liposomal hydroxycamptothecin (CLH) and 5-aminolevulinic acid (5-ALA). CLH was composed of soybean lecithin, cholesterol, and octadecylamine and was prepared using a thin-film method. After carrying out in vitro studies, it was observed that Chemo-SDT was more cytotoxic against the tumor cells as compared to intratracheal or intravenous administered cationic liposomal hydroxycamptothecin (CLH) and SDT alone. In vivo studies were carried out on metastatic lung tumor-bearing mice. It was observed that the most effective form of therapy was the inhaled combined Chemo-STD therapy. The single therapy (both intravenously and intratracheally) and SDT treatment options weren’t found to be as effective. The success of this was attributed to the synergistic effect of both chemotherapy and SDT in improving cancer cell apoptosis [72].

5-Fluorouracil has been widely studied as an anticancer agent. 5-FU can be administered as an IV bolus or IV infusion; however, these lead to toxicity in the body [73]. Therefore, a liposomal formulation was made in order to maximize concentration at the tumor site in the lungs while minimizing concentration in healthy tissues. 5-Fluorouracil was incorporated into different liposome formulations using the thin film hydration method and the sustained release rate was studied. The dipalmitoyl phosphatidylcholine (DPPC) liposome formulation had a diameter of 1.7 ± 1.3 μm while dipalmitoyl phosphatidylglycerol (DPPG) had a vesicle diameter of 2.6 ± 1.0 μm. These inhibited liposome aggregation and fusion, supporting the formulations’ stability. Dipalmitoyl phosphatidic acid (DPPA) formulations had the best release rate constant (k) of 0.99 h^−1^ with the lowest vesicle diameter of 0.4 ± 0.2 μm. Therefore, liposomal formulations can be created to allow for a sustained release of drugs into the lungs and thereby decrease the frequency of administration [73].

##### Nanoemulsions

An emulsion is a colloid that is composed of two immiscible liquids, often oil and water, with one of the components disseminated in the other. The two phases in emulsions are (i) the dispersed phase consists of the liquid that makes up the droplets, and (ii) the continuous phase being the liquid that surround the droplets [74]. Therefore, two main types of emulsions can be formed, (i) water-in-oil type, with water droplets dispersed in the continuous oil phase and (ii) oil-in-water, where oil is the dispersed phase in the aqueous continuous phase [75].

**Figure 5:**
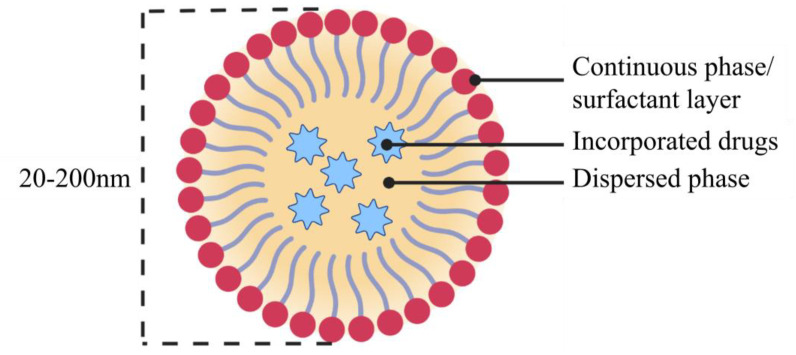
Schematic representation of an oil-in-water nanoemulsion.

Nanoemulsions (NEs) are hence transparent or translucent, heterogeneous dispersions of the two phases, stabilized by the interfacial layer of a surfactant, usually having a mean average diameter of 20–200 nm, possessing high kinetic and thermodynamic stability [76,77,78]. Their structure is illustrated in Figure 5.

Nanoemulsions are gaining popularity as drug carriers, since they are made from surfactants that have been approved for human consumption that are “Generally Recognized as Safe” (GRAS) by both the US FDA (“Food and Drug Administration”) and the EPA (“Environmental Protection Agency”) [79]. The superiority of nanoemulsions over conventional forms of therapy comes from their capability to dissolve large quantities of hydrophobic drugs within their lipophilic core. They also prevent the enzymatic degradation and hydrolysis of loaded drugs [80]. This helps in reducing drug administration frequency and allows for a sustained drug release mechanism. Furthermore, because of their small size and ability to overcome cell barriers via diffusion, NEs can maintain pulmonary retention and deposition in lung tissue for prolonged periods of time. Another advantage of nanoemulsions is that they can be easily used for medication co-loading [81,82].

Because NEs act similarly to solutions, when nebulized, the formulations display significantly improved in vitro aerosolization performance as compared to dry powders [83,84]. Therefore, instead of solid dry powder formulations, all NEs developed were aerosolized using nebulizers.

The composition of the dispersed and continuous phase of nanoemulsions also impacts its pharmacological properties. Nanoemulsions can be built using long, medium, short-chain, or a combination of either fatty acid as the dispersed phase. However, it was noted that the nontoxic formulations of nanoemulsions used in intratracheal delivery of chemotherapeutics are usually composed of a mixture of medium and long chain fatty acids [85]. The surfactant layer is also important in tuning the properties of the NEs for pulmonary delivery. Low molecular weight and ionic surfactants, such as sodium lauryl sulfate, are commonly utilized. Nonetheless, ionic surfactants have been discovered to be toxic and damaging to biological membranes [86]. Ionic surfactants are also not as effective as lipophilic non-ionic surfactants in dissolving drugs. Hence, the use of ionic surfactants is not very common. Additionally, non-ionic surfactants are adept emulsifiers, useful in self-emulsifying drug delivery systems and can enhance the size, shape, and stability of the particles [87]. Lastly, synthetic emulsifiers could lead to an adverse reaction between the nanoemulsions and lungs leading to enzyme disfunction and alteration of protein structure and lipid bilayers in the membrane [88]. Consequently, replacing synthetic surfactants with natural substitutes have a greater potential for application.

Naringin and celecoxib were co-loaded onto a nanoemulsion of oil in water [89]. Celecoxib is known to be used in targeted cancer therapy and causes tumor growth reduction in some lung cancer models, and the herbal flavanone glycoside compound derived from naringin is reported to have some anticancer activity against lung cancer. Elbahr et al. used homogenization/solvent evaporation to create two nanoemulsions—F1 and F2—with ethanol as a co-surfactant alcohol [90]. F1 and F2 particle sizes were 75.48 ± 0.62 and 106.20 ± 1.88 nm, respectively, which is suitable for both passive cancerous cells targeting and evading macrophagic phagocytosis clearance. The TEM images showed spherical well dispersed nanoemulsion particles. An MTT cytotoxicity assay of F2 on A549 cell lines was carried out that showed dose-dependent toxicity and higher toxicity as compared to the combination of naringin and celecoxib in plain DMSO. The nano-size of nanoemulsions allows for better cellular internalization via endocytosis, increased membrane permeability, and cellular internalization of poorly lipid-soluble anticancer drugs into cancer cells. When aerosolized, formulation F2 was found to be highly stable with and aerodynamic diameter of 101.22 ± 1.31 nm and displayed good biodistribution [89].

Asmawi et al. developed docetaxel NEs with biocompatible excipients for pulmonary drug delivery [90]. This was designed to overcome docetaxel’s low solubility and to enhance its bioavailability and efficacy. A high-energy emulsifying technique was used to create the nanoemulsion formulation. The oil phase consisted of a mixture of medium- (lauric fatty acids and palm kernel oil esters) and long-chain fatty acids (myristic fatty acids) and a non-ionic hydrophilic surfactant—lecithin was mixed with the oils. Lecithin is non-toxic, biocompatible, and pH insensitive, hence a very good choice for nanoemulsion formulations [91]. The morphology of the prepared docetaxel-loaded NE formulation was found to be spherical-shaped with a particle size of 94.35 ± 0.77 nm and 100% entrapment efficiency. The optimized NEs were aerosolized using an OMRON MicroAIR nebulizer and showed desirable aerosolization properties with an aerodynamic diameter of 3.02 ± 0.26 μm, FPF of 92.76 ± 0.63%, and was deemed suitable for pulmonary delivery. In vitro cell culture studies revealed that the developed formulation inhibited the proliferation of A549 cells (human lung carcinoma cells) while having no effect on normal cell proliferation (MRC-5). The created NEs were found to be promising carriers for docetaxel in the treatment of lung cancer using inhalable formulations. [90].

##### Solid Lipid Nanocarriers

Solid lipid nanocarriers (SLN) are nanoemulsions in which the liquid dispersed phase is replaced with a liquid matrix that is solid at room temperature and has a submicron colloidal size between 50 and 1000 nm [92]. Triglycerides (e.g., tripalmitin), partial glycerides (e.g., glyceryl behenate), fatty acids (e.g., decanoic acid), waxes (e.g., cetyl palmitate), and steroids (e.g., cholesterol) are among the most commonly used solid lipids [93]. The use of solid lipids rather than oils aids in the achievement of controlled drug release. This is possible due to the lower drug mobility in solid lipids than in oily ones. Hence, SLNs possess the advantage of liposomes (safe, non-toxic), are physically stable, and hold the possibility of large-scale production. However, one drawback of SLNs is their low drug-loading capacity and drug expulsion during storage. They are typically synthesized using high-pressure homogenization (HPH) or micro-emulsification techniques. The drug becomes trapped within the lipidic matrix. [92,94].

Studies have shown that solid lipid nanocarriers are localized in the bronchial area (upper respiratory tract) of the lungs from where they get cleared primarily via the mucociliary escalator [95]. Some larger SLNs have also been found in the pulmonary region of the lungs, resulting in longer lung retention time [96].

Nassimi et al. conducted a study to assess the toxicological therapeutic window for solid lipid nanoparticles (SLNs) as a pulmonary drug delivery system [97]. To do so, they developed blank SLN formulations using a high-pressure homogenization method. The lipid matrix of the SLN was composed of a triglyceride (Softisan^®^) and a phosphatidylcholine (Phospholipon^®^ 90G). The surfactant used was Solutol^®^HS15 along with distilled water. The resulting solid lipid nanocarriers were characterized with a hydrodynamic diameter of 98.4 ± 4.9 nm and a polydispersity index (PDI) of 0.148. An MTT cytotoxicity assay and neutral red uptake assay (NRU) were carried out on the A549 cell lines. The cells were treated with the blank SLN formulation for 24 h, and it was observed that the SLN had a concentration-dependent reduction in A549 cell viability. The WST-1 assay was carried out on murine precision-cut lung slices (PCLS). They were exposed to the SLN for 24 h and their metabolic activity was found to be reduced. Lastly, an in vivo cytotoxicity assay was carried out on female BALB/c mice where they were exposed to the SLN for 16 days. There was not any increase in lactate dehydrogenase (LDH) levels, suggesting minor damage to the cell membranes, suggesting the SLN was not cytotoxic to the lung tissues. No other signs of inflammation such as chemokine KC, IL-6, or neutrophilia were observed either. These observations indicated no toxicity of SLNs when delivered intratracheally at concentrations lower than a 200 μg [97].

Epirubicin, an anthracycline and a stereoisomer of doxorubicin, is known to show anticancer properties but has limited use due to the adverse side effects associated with it. To improve the stability and make it safer, solid lipid nanoparticle formulation of epirubicin was made [98]. The SLNs were composed of soy lecithin, Compritol 888 ATO^®^, and Poloxamer 188^®^. The particle size, zeta potential, and percent entrapment efficiency of the produced SLNs were determined. They were found to be 223.7 nm, −30.6 mV, and 78.9%, respectively. To allow for intratracheal administration, the SLNs were nebulized using Pari Inhalierboy (Starnberg, Germany). The blank SLNs, epirubicin-loaded SLNs and pure epirubicin solution showed respirable fractions (RF) of 77.03%, 78.46%, and 59.51%, respectively, indicating the lowest level of drug loss in epirubicin-loaded SLNs. SLNs were also successful in deep lung delivery of the drug. In vitro cytotoxicity assay was carried out using A549 cell lines. The developed formulation was found to be more cytotoxic than the free drug against A549 cancer cells. An in vivo assay was carried out on infected male Sprague–Dawley (SD) rats, the results of which indicated better lung deposition of epirubicin when administered as the aerosolized SLN formulation as compared to simple epirubicin solution. Pharmacokinetic studies also revealed a 2.07-fold higher plasma area under the curve values for epirubicin-loaded SLNs. This data suggests that inhalable SLNs is a suitable formulation for intratracheal delivery to treat LC [98].

Paclitaxel (PTX) and curcumin (CU), as discussed previously, have anticancer properties; however, factors such as poor solubility and low bioavailability make their use limited. A combination regimen of PTX and CU using solid lipid nanoparticles (SLNs) was designed to improve their therapeutic potential. The authors were able to successfully co-encapsulate PTX and CU in SLNs (PC-SLNs) with high encapsulation efficiency (CU: 97.6%, PTX: 95.8%). The SLNs were characterized and found to have a particle size, PDI, and zeta potential of 121.8 ± 1.69 nm, 0.267 ± 0.023, and –30.4 ± 1.25 mV, respectively [99]. These SLNs had an improved area under the curve, longer lung residence time, and increased the half-life, thereby increasing the circulation time. PC-SLNs showed a better inhibitory effect than PTX alone or the unformulated combination of CU and PTX in the in vitro studies carried out on A549 cell lines. It was also observed that PC-SLNs could be administered in lower doses while also maintaining the therapeutic effect. For the purpose of in vivo studies, a nude mouse xenograft tumor model was used. PC-SLNs showed promising results with a tumor suppression rate of 78.42%, compared to 40.53% for PTX and 51.56% for CU + PTX. Because of the synergistic effect of PTX and CU, PC-SLNs have high clinical therapeutic value for lung cancer treatment. These findings indicate that drug combination therapy with solid lipid nanocarriers is extremely promising for the treatment of LC [99].

##### Nanostructured Lipid Carriers

Nanostructured lipid carriers (NLCs) are an advanced type of solid lipid nanoparticle. SLNs were known to have (i) poor loading capacity, (ii) drug expulsion once stored, and (iii) high water content (70–95%) in aqueous SLN dispersions. These were overcome by NLCs owing to a less defined solid lipid matrix formed by controlled addition of lipids [92,100,101]. The solid matrix of NLCs contains tiny liquid nano-compartments of oil (Figure 3). The drug’s solubility is higher in these oil compartments, increasing the total drug loading capacity. The procedure of synthesizing NLCs requires mixing a large volume of liquid lipid (oil) with a solid lipid. When the concentration of oil is low, it becomes evenly distributed in the solid. As concentration increases, the solid becomes saturated with oil and a phase separation is observed. This leads to the formation of oily nano-compartments in the solid lipid [102,103]. They have an average size of 200 nm. NLCs that are formed from *o*/*w* emulsion processes can be used to carry a wide variety of drugs ranging from siRNA, natural compounds, and drugs with varying physicochemical properties. However, NLCs have one drawback. An organic solvent is required to load hydrophobic drugs due to their low solubility in NLCs [104,105,106].

An NLC formulation of paclitaxel was employed for intratracheal tumor targeted delivery by Jenning et al. NLCs were synthesized using the melted ultrasonic method. The solid lipid used was Precirol ATO 5^®^, squalene was used as the liquid lipid, and soybean phosphatidylcholine was employed as the emulsifier. The aqueous phase was composed of a surfactant called Tween 80^®^ and (N-[1-(2,3-dioleoyloxy)propyl]-N,N,N-trimethylammonium) “DOTAP” (a cationic lipid that grants positive charge to NLC) in deionized distilled water. Prior to loading, the drug was solubilized in DMSO. To link the LHRH peptide, PEG2000 was employed. Lastly, it was conjugated with siRNA. The resulting formulation was abbreviated LHRH-NLC-siRNAs-PTX. The morphology of the hence formed particles was found to be spherical. LHRH-NLC-siRNAs-PTX had a size of 113 nm and a high entrapment efficiency of 98%. The in vitro cytotoxicity assay revealed a higher toxic effect of the NLCs than gemcitabine against A549 lung cancer cell lines. The formulation was administered via IV and inhalation (using a Collison nebulizer BGI Inc., Waltham, MA, USA) routes in the in vivo study using an orthotopic NSCLC mouse model. When administered via the pulmonary route, the formulation was found to have significant lung accumulation and retention. According to the immunoperoxidase assay, the formulation did not elicit an immune response in human peripheral blood lymphocytes. Furthermore, no in vivo toxicity was observed in any major organs such as the liver, kidney, spleen, heart, lung, and brain of nude mice after either intratracheal or intravenous administration [103].

##### Lipid–Polymer Hybrid Nanocarriers

**Figure 6:**
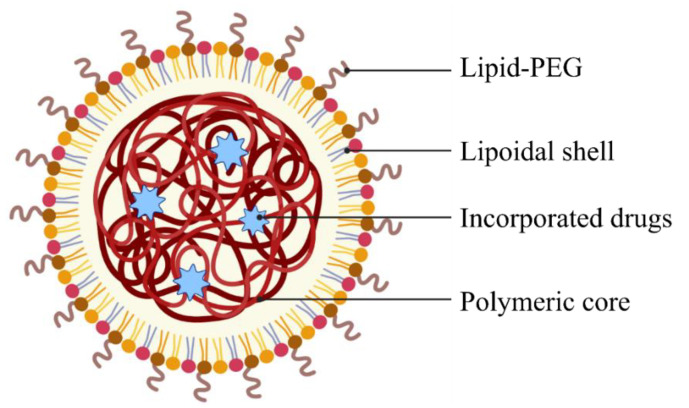
The structure of lipid polymer hybrid nanoparticles with inner polymer core containing drug molecule surrounded by lipid and lipid PEG layer.

Lipid-based nanoparticles can be further modified by combining them with polymers to form lipid–polymer hybrid nanoparticles (LPHNPs). These novel drug delivery systems are successful in merging the advantages of polymeric as well as lipid-based nanocarriers. The polymer core provides structural integrity and physical stability while the liposomal shell is biodegradable, provides enhanced cellular uptake and cell affinity, elevated biocompatibility, and bioavailability. Finally, it is coated with a layer of lipid–PEG. This coating serves two main purposes: (a) it acts as a stealth coating and allows for a longer circulation time in vivo, and (b) provides steric stabilization. In addition, the inner liposomal shell prevents any leakage of drugs and provides efficient drug encapsulations. The detailed structure of a lipid–polymer hybrid nanoparticle is illustrated in Figure 6 [107,108,109].

Bardoliwala et al. developed an inhalable dry powder formulation containing polymeric lipid–polymer hybrid nanocarriers (LPHNCs) loaded with docetaxel [110]. Because docetaxel has a low water solubility, it must be formulated with a nonionic surfactant and ethanol to improve solubility. This frequently causes hypersensitivity in patients taking the drug. As a result, two designs of LPHNs were developed to overcome these disadvantages: Plackett–Burman design (PBD) and Box–Behnken design (BBD). The optimized LPHN formulation was lyophilized in a Virtis advance lyophilizer using various cryoprotectants to increase drug retention. To optimize aerodynamic properties, a dry powder inhaler was constructed using various grades of course and fine lactose. Docetaxel release was also increased in the LPHNs developed. These LPHNs had a fine particle fraction (FPF) of 68.3 ± 2.5% and a high drug retention of 98.3 ± 3.1%. Hence, the created LPHNCs have potential to be optimized into a potential anticancer agent for treatment of LC through the pulmonary route [110].

**Table 2:** Inhalable lipid nanocarriers for drug delivery against LC.

Drug	Lipid-Based Carrier	Composition	Key Outcome	Refs.
9-Nitrocamptothecin (9NC)	Liposome	Dilauroylphosphatidylcholine (DLPC)	The dose of aerosolized L-9NC was 3–20 times lower than the intramuscular and intravenous doses of 9NC.	[111]
Camptothecin (CPT)	Liposome	Dilauroylphosphatidylcholine (DLPC)	The highest drug concentrations in the lungs were obtained with the inhalable CPT liposome aerosol.	[112]
Paclitaxel	Liposome in bacteria	Soybean lecithin and cholesterol, *E. coli*	Highest anticancer effect, with VEGF and HIF-1 downregulation and improved cancer cell apoptosis.	[113]
Paclitaxel	Liposome	Dilauroylphosphatidylcholine (DLPC)	The AUC in the aerosol group was 26 times higher than the IV injection group.	[71]
Curcumin	Liposome	Poloxamer 188, 2-hydroxypropyl—cyclodextrin, lecithin, cholesterol, stearylamine	Liposome formulation outperformed curcumin powder in terms of rate and extent of lung tissue absorption, as well as mean residence time within lung tissues.	[114]
Curcumin	Liposome	Soybean lecithin and cholesterol	Liposomal curcumin dry powder demonstrated superior anticancer activity and selectivity over free curcumin.	[68]
Doxorubicin	Transferrin-conjugated liposome	DSPC, DSPE-PEG2000, DSPE-PEG-COOH, and cholesterol	The group of animals treated with TF-liposomes lived longer than those in the other treatment regimens, and tumor size was reduced.	[115]
Vincristine	Liposome	Soy phosphatidylcholine and cholesterol	In comparison to the free drug, the developed formulation had improved pharmacokinetic behavior, with increased maximum concentration and systemic exposure and decreased elimination half-life.	[116]
Docetaxel	Nano emulsion	Lauric fatty acids. Myristic fatty acids, PKOE, lecithin, Tween 85^®^, Span 85^®^, and glycerol	Good inhalable formulation for administration of docetaxel. Human lung carcinoma cell (A549) is more selective than normal cell (MRC-5).	[90]
Docetaxel and Curcumin	Nano emulsion	PKOE, lauric FA, myristic FA, lecithin, Tween 85^®^, Span 85^®^, and glycerol	Docetaxel and curcumin had a synergistic anticancer effect, and the nanoemulsion had desirable physicochemical and aerodynamic properties for pulmonary delivery.	[117]
Blank formulation	SLN	Lipid mixture (Softisan^®^ and Phospholipon^®^ 90G) and Solutol^®^ HS15 as surfactant.	Blank SLNs are adequate for pulmonary delivery of drugs, intratracheally. There are no side effects or indicators of cell death or inflammation.	[97]
Erlotinib	SLN	Compritol 888 ATO^®^, Tween 80^®^, poloxamer 407^®^.	The created erlotinib loaded SLNs outperformed free erlotinib in terms of cumulative drug release profile and anticancer activity.	[118]
Myricetin	SLN	Gelucires (G 39/01, 50/13, 44/14) and compritol	The use of gelucire-based SLNs was proven to improve the drug’s physiochemical properties, release, and anticancer effects.	[119]
Paclitaxel	NLC	Precirol ATO 5^®^, squalene, Tween 80^®^	Inhaled NLCs had the highest drug concentration in the mice’s lungs, with no signs of systematic cytotoxicity when contrasted to the IV route.	[120]
Paclitaxel	NLC	Stearic acid (or glyceryl monostearate) oleic acid, Tween 80^®^, Tween 20^®^, or Tween 40^®^	When compared to free drug treatment, inhaled paclitaxel-NLCs demonstrated favorable organ distribution and superior anticancer effect.	[121]
Celecoxib combined with IV docetaxel	NLC	Compritol^®^, miglyol^®^, and sodium taurocholate	The synergistic effects of celecoxib-NLCs inhalation and IV docetaxel were demonstrated in vivo.	[122]
siRNA	LPHNs	Poly(lactic-co-glycolic) acid and Dipalmitoylphosphatidylcholine	Inhibition of ENaC protein expression in A549 cell lines for an extended period of time was observed. In vitro aerosol performance was found to be optimal after using a vibrating mesh nebulizer for delivery.	[123]
Gemcitabine and cisplatin	Niosomes	Tween 65^®^, Span 60^®^, cholesterol, sodium dodecyl sulfate (SDS), glycerol	Developed NGCs had a lower cytotoxicity effect against MRC5 as compared to the free drug.	[124]

#### 4.1.4. Gelatin-Based Nanocarriers

Gelatin is a protein that has been denatured by acid or alkaline hydrolysis of animal collagen. Gelatin is an excellent choice due to its biocompatibility and biodegradability. Gelatins also have cationic, anionic, and hydrophobic amino acid groups in a 1:1:1 ratio. This makes a large variety of functional groups on the gelatin nanoparticle surface available for conjugation with ligands, such as carboxyl, hydroxyl, and amino groups. A tumor-specific ligand may be used to deliver drugs to the desired tumor cells in a specific location. This will avoid toxicity to the normal healthy cells and increase drug concentration at tumor cells. Various studies have confirmed stability of aerosolized gelatin nanoparticles with optimal aerodynamic properties for pulmonary delivery [125,126,127,128].

Tseng et al. used gelatin nanoparticles (GPs) as carriers of cisplatin (CDDP) to enhance its therapeutic effect and minimize side effects [129]. The CDDP gelatin nanoparticles (GP-Pt) were coated with biotinylated epidermal growth factor (bEGF). This formulation was abbreviated as GP-Pt-bEGF. Because of its selectivity for epidermal growth factor receptor, the GP-Pt-bEGF modification resulted in localized delivery to A459 human lung cancer cells with no selectivity for human fetal lung (HFL1) cells. In an in vitro anticancer study, GP-Pt-bEGF was found to be more potent than free CDDP or GP-Pt due to its faster effect on the cell cycle and lower IC_50_ for inhibiting A549 cell growth. An in vivo assay was carried out where SCID mice were administered with free CDDP, GP–Pt, and GP–Pt–bEGF via intratumoral injections. It was discovered that GP-Pt-bEGF had greater antitumor activity while being less toxic to healthy cells than free CDDP. When the same formulations were administered via the pulmonary route, it was observed that GP–Pt–bEGF could specifically target cells overexpressing for EGFR. This means a higher concentration of cisplatin could be achieved in tumor cells. Hence, gelatin-based nanoparticles are a promising carrier for achieving highly selective delivery of drugs into tumor cells [129].

#### 4.1.5. Inorganic Nanocarriers

Magnetic nanoparticles (MNPs) are being studied for their potential use in site-specific drug delivery to the pulmonary system. However, if magnetic nanocarriers are not transported properly, they may cause toxicity in the lungs. To avoid this, the particles must be extremely sensitive to electromagnetic fields and capable of traveling long distances [130,131].

Because of their large pore volume and surface area, mesoporous silica NPs (MSNs) have also been used for targeted drug delivery [132]. These characteristics allow for the efficient loading of hydrophobic chemotherapeutics. They can be entrapped in pores to protect themselves from degradation, or be conjugated to available surface functional groups, particularly silanol groups, via covalent or electrostatic interactions. MSN surfaces can be functionalized with receptor-specific proteins to ensure target-specific delivery. They can also be coated with nanoparticles or polymers to enhance controlled release of drugs and stability [133].

Taratula et al. created a tumor-targeted mesoporous silica nanoparticles (MSN)-based drug delivery system capable of delivering chemotherapeutic drugs such as doxorubicin and cisplatin using two types of siRNA targeted to NSCLC MRP1 and BCL2 mRNA. To target the specified mRNA in tumor cells, a luteinizing hormone-releasing hormone peptide was conjugated onto the MSN’s surface. The preferential accumulation of the formulation in the mouse lungs resulted in increased cytotoxicity. This also prevented MSN from entering systemic circulation, limiting their accumulation in other organs [134].

Zinc oxide nanoparticles (ZnO-NPs) have unique characteristics such as tunable surface and particle size, chemical inertness, and desirable biocompatibility owing to the fine particle size, the specific surface area and molecular arrangement, and electronic structure. ZnO-NPs have also been shown to cause tumor death, decrease drug resistance, and minimize side effects in vitro, when taken in combination with chemotherapeutics [135]. Umamaheswari et al. used a green synthesis method for the production of ZnO nanoparticles [136]. Raphanus sativus var. Longipinnatus leaves were obtained from the fresh plant, boiled, and then filtered. The filtrate was centrifuged, and the supernatant was used for ZnO nanoparticle synthesis along with zinc acetate. The biosynthesized ZnO NPs were spherical in shape and had an average size of 209 nm and a zeta potential of -13.7 mV. An in vitro MTT assay was carried out using A549 cell lines and it was found that the biosynthesized ZnO nanoparticle led to an enhanced cytotoxic effect [137].

Hu et al. developed ZnO-NPs and loaded them with cisplatin (Cp) (ZnO-NPs(Cp)), gemcitabine (Gem) (ZnO-NPs(Gem)), and a combination of both (ZnO-NPs(Cp/Gem)) [135]. ZnO-NPs(Cp/Gem) were found to be stable and spherical in shape with size in the order of ~20 nm. An MTT cell viability assay was performed to test the activity of the hence-produced NPs. This assay showed a significant increase in cytotoxicity against A549 cells when treated with ZnO-NPs(Cp/Gem) as compared to unformulated Cp, Gem, or their combination (Cp + Gem). Moreover, ZnO-NPs(Cp/Gem) significantly enhanced Cp and Gem’s apoptosis-promoting effect in A549 cells. Therefore, ZnO-NPs(Cp/Gem) are highly promising carriers for loading chemotherapeutics [135].

Silibinin is a flavonolignan derived from the Silybum marianum (milk thistle) plant’s silymarin extract and is known to inhibit angiogenesis, cell proliferation, drug resistance, and metastasis in lung cancer models [138,139]. Yet, silibinin’s therapeutic use is limited by its low aqueous solubility, poor penetration, and increased metabolism leading to rapid systemic clearance. Hence, Ravi et al. prepared silibinin-loaded gold nanoparticles (Sb-GNPs) using the Turkevich and Frens method with a citrate reducing agent and stirring the prepared gold nanoparticle with a silibinin solution [136]. GNPs synthesized were discovered to be monodispersed and spherical in shape. The silibinin was successfully conjugated with gold nanoparticles, and FTIR and DLS confirmed the long-term stability of GNP and Sb-GNP nanoconjugates in suspension phase. An in vitro MTT assay was performed on A549 cell lines to measure the efficiency of Sb-GNPs. Cell viability was found to be lower when treated with Sb-GNPs than when treated with free silibinin. Trypan blue dye exclusion assay showed that to produce a ~35–40% decrease in cell viability, a 5 μM dose of Sb-GNP was required in contrast to a 25 μM dose of free silibinin. Hence, it was concluded that conjugated silibinin was 4–5 times more efficacious than unconjugated [136].

Vera-Nuñez et al. carried out biosynthesis of a novel silver nanoparticle conjugated with Thelypteris glandulosolanosa (raqui-raqui) [140]. Silver nitrate (AgNO_3_) was reduced and stabilized using raqui-raqui aqueous extract to form Ag nanoparticles (AgNPs-RR). They were characterized using dynamic light scattering and scanning transmission electron microscopy and found to be spherical in shape with a mean hydrodynamic size equal to 39.16 nm. Upon carrying cell toxicity studies in A549 cells, the biosynthesized NP exhibited significant antitumor activity with an IC_50_ value of 12.50 µg/mL [140]. 

He et al. demonstrates the antitumor efficacy of Ag nanoparticles synthesized via green route against lung cancer both in vitro and in vivo [141]. Cytotoxicity effect was shown on human lung cancer H1299 cells. This formulation showed dose-dependent cytotoxicity and stimulation of apoptosis in H1299 cells. The effects on H1299 cells correlated well with the inhibition of NF-κB activity, a decrease in bcl-2, and an increase in caspase-3 and survivin expression. This study found that it has a significant inhibitory effect on the proliferation of NSCLC cells (H1299). In vivo studies employing these nanoparticles on a mouse xenograft tumor model showed considerable effectiveness in reducing the development of H1299 tumors in severe combined immunodeficient (SCID) mice.

Another form of tumor ablation for lung cancer is magnetic hyperthermia, which is noninvasive and uses heat generated by magnetic materials. Superparamagnetic iron oxide (SPIO) is a potential hyperthermia agent and drug delivery carrier [142,143]. Sadhukha et al. carried out a study to assess the efficacy of SPIO nanoparticles as hypothermia agents [143]. The NPs were synthesized using iron chlorides and ammonium hydroxide. SPIO NPs were characterized using dynamic light scattering and were to have an average hydrodynamic diameter of s 309 ± 24 nm. Aerosolization was carried out using ultrasonic atomization with an MMAD of 1.1 ± 0.1 mm. It was observed upon inhalation, targeted SPIO particles caused a significant decrease in lung tumor size [143].

#### 4.1.6. Micelles

Micelles are self-assembling nano-sized colloidal particles, with size ranging 5–100 nm. Micelles consist of a dispersed phase, usually particulate matter, distributed inside a dispersion medium, which is essentially a continuous phase. They are formed from amphiphilic block copolymers or surfactants [144].

**Figure 7:**
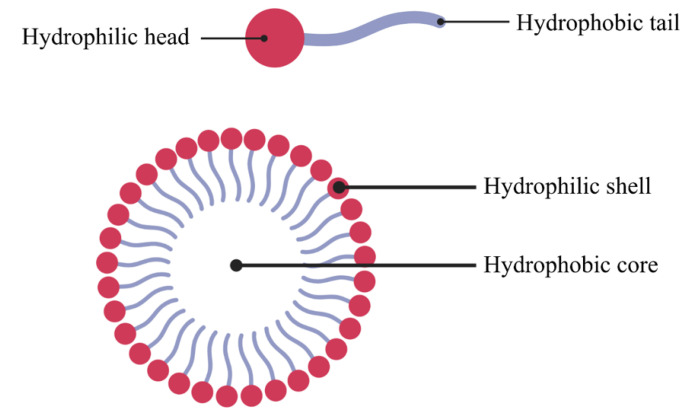
Labelled structure of a micelle.

Micelles exist as monomers in aqueous medium at low concentrations. When concentrations exceed the critical micellar concentration (CMC), they self-assemble into micelles, which have a spherical shape, a hydrophobic core, and a hydrophilic shell (Figure 7). The aggregation number of micelles is defined as the number of individual molecules coming together to form a micelle [145].

Micelles can solubilize a wide range of compounds with varying solubilities in their core. The polarity of the drug influences solubilization and location. Solubilization of hydrophobic drugs in the micelle core is facilitated by lengthening the hydrophobic chain length. Micelle loading efficacy towards hydrophobic drugs ranges between 5 and 25% by weight [10]. Micelles are also capable of enhancing permeability across physiological barriers and avoiding lung clearance by alveolar macrophages owing to their small size and hydrophilic shell. Due to these reasons, micelles can allow sustained drug release and are good carriers of drugs for pulmonary delivery [10,144,146,147]. Micelles’ composition varies depending on their application. Micelles’ lipophilic cores can be formed with phospholipids or hydrophobic polymers, whereas the hydrophilic core is typically composed of PEG. The latter has a molecular weight that ranges from 1 to 15 kDa. Endogenous lung phospholipids, such as diacylphosphatidylethanolamine, a component of the lung surfactant, can be used to create a carrier suitable for targeted pulmonary drug delivery [144].

Rezazadeh et al. synthesized paclitaxel (PTX) loaded novel mixed polymeric micelles based on tocopheryl succinate polyethylene glycol (PEG) 1000 (TPGS1K) and 5000 Da (TPGS5K). The micelles were then aerosolized using the spray-dry technique to form respirable micron-size particles with a narrow particle distribution and a spherical particle morphology. The particle size of spray-dried powders was 2.5 μm. The two polymeric micelles, TPGS5K and TPGS1K, were mixed together in different ratios and their CMCs were measured. TPGS5K/TPGS1K (7:3) and TPGS5K/TPGS1K (5:5) were found to have CMCs of 17.89 and 16.33 μM, respectively. A cytotoxicity assay was performed on human lung cancer A549 cells, and it was discovered that PTX-loaded mixed micelles had higher cytotoxic activity than the free drug. Spray drying of PTX-loaded micelles with lactose resulted in the production of inhalable powders with a high fine particle fraction (60%), according to the in vitro deposition data. Inhalable, dry powder, PTX-loaded micelles have great application in pulmonary delivery for lung cancer treatment [148].

#### 4.1.7. Aptamers

The term “aptamer” comes from the Latin word “aptus,” which means “adapted” or “conformable.” Aptamers are single-stranded RNA or DNA oligonucleotides with a high level of affinity for organic compounds (including proteins) or inorganic molecules [149]. They are also termed as “chemical antibodies”. They have higher reaction specificity, lower molecular weight, lower costs of production, and lesser variability in the stage of production when compared to monoclonal antibodies. Aptamers contribute to the immunogenicity and specificity of nanoparticles, whereas nanoparticles extend the half-life of aptamers in plasma and balance their clearance [150]. In addition, aptamers, when complexed with chemotherapeutic agents, lower the latter’s toxicity and help eliminate severe side effects. Hence, by combining nanocarriers and chemotherapeutic drugs labeled by aptamers, a promising treatment strategy can be formulated that may lead to prolonged and specific drug release at the tumor site [150].

Ma et al. prepared a novel aptamer modified CD133 docetaxel (DTX) liposome system in order to investigate its characteristics in vitro and in vivo [151]. The CD133^+^ antigen is known to be a marker of lung cancer stem cells (CSCs), while aptamer A15 is a promising ligand of the former [149,152,153]. Thus, the A15-CD133 interaction could help facilitate effective drug delivery to CD133 positive lung CSCs. The DTX containing liposomes were synthesized using a thin-film hydration method. The conjugation of the CD133 aptamers to DTX liposomes was carried out by a thiol–maleimide reaction. The hence produced CD133 aptamers modified DTX liposomes (CD133-DTX LP) were found to be sub-spherical in shape and aggregated to form small clusters. They had a particle size of 116.5 ± 9.3 nm and an entrapment efficiency of 88.6 ± 7.1%. A slow release of DTX was observed from the formulation in vitro, possibly due to a hindrance caused by the CD133 aptamer. CD133 aptamers modified DTX LP significantly decreased cell proliferation and improved therapeutic efficiency in a cytotoxicity study. The in vivo imaging results showed that CD133-DTX LP had excellent tumor targeting ability. In vivo antitumor activity revealed that the CD133-DTX LP exhibited significant antitumor activity in A549 tumor mice while causing extremely low systemic toxicity [151].

Wu et al. developed an aptamer-decorated hybrid nanoparticle for the co-delivery of docetaxel prodrug (DTXp) and cisplatin (DDP) for treatment of lung cancer [154]. The aptamer was conjugated onto PLA-PEG-Mal polymer to form the aptamer conjugated lipid polymer hybrid nanoparticle (LPHN). The aptamer modified LPHNs were co-loaded with DTXp and DDP (APT-DTXp/DDP-LPHNs) using the thin-film hydration method along with the ultrasonic dispersion method. APT-DTXp/DDP-LPHNs were found to have a particle size of 213.5 ± 5.3 nm, with a zeta potential of 15.9 ± 1.9 mV. This modified polymer had a higher uptake by A549 cells as compared to the unmodified version. An MTT assay was carried out to evaluate cell viability of the formulation and it was observed that unloaded APT-LPHNs were non-toxic to A549 cells. APT-DTXp/DDP-LPHNs had an IC_50_ of 0.71 ± 0.09 μg/mL, showing greater toxicity against A549 cells than unmodified LPHNs. And the synergic antitumor effect of DTXp and DDP was evaluated using a combination index analysis, which was measured to be 0.62. Hence, the formulated nanocarrier is of great potential due to the synergic effect of DTXp and DDP, along with the aptamer modification of LPHNs [154].

### 4.2. Microparticles

Microparticles (MPs) usually have an average mean size of 5 μm and can aggregate into particles inside a dry powder inhaler. Their large geometric size leads to ease of particle dispersion. Hence, they can easily be aerosolized in the form of dry powder formulations [155,156]. With an aerodynamic diameter of >5 μm, microparticles can effectively escape impaction in upper airways and penetrate deep into the lungs. Owing to their large size, microparticles are also effective in escaping phagocytosis by alveolar macrophages and may therefore help achieve sustained release pulmonary administration formulations [157,158,159]. In order to achieve sustained drug release, synthetic hydrophobic polymers are commonly used in the fabrication of MPs. Spray-drying can successfully produce inhalable PLGA, PCL, or PLA MPs [73]. Other than PLGA, other polymers such as alginate, chitosan, or poly(glycerol adipate-co-ω-pentadecalactone), a biodegradable polyester, as well as lipids as dipalmitoylphosphatidylcholine, tristearin, Compritol and glyceryl behenate can be used to achieve sustained drug release within the lungs [160,161,162,163,164,165].

Shepard et al. created a dry powder pulmonary formulation of bevacizumab using the spray drying technique [166]. It is an intravenous monoclonal antibody approved for the treatment of non-small cell lung cancer (NSCLC) [167]. However, bevacizumab therapy is fraught with complications because it is administered systemically in high doses and is costly. During spray drying, collapsed spherical particles of bevacizumab were formed, with the majority of particles measuring ~1 to 5 μm in diameter. The size of the aerodynamic particles was 2.0 ± 1.6 μm. The mechanism of action of bevacizumab is to inhibit VEGF expression in cancer cells. An anti-VEGF activity assay revealed that forming bevacizumab into a dry powder had no effect on its activity. In an in vivo rat model for NSCLC, the formulation was found to be effective at a 10-fold lower dose than the intravenous control. As a result, by reformulating bevacizumab for local delivery, side effects, dose reductions, and improved patient compliance are all possible [166].

### 4.3. Nanocomposites and Nanoaggregates

Nanocarriers, despite being good carriers of drugs have a size of <0.5 μm and hence could easily be exhaled out of the lungs before reaching the tumor site. The nano size also makes loading into devices such as dry powder inhalers difficult. The microparticles, despite overcoming this challenge, is posed with lung clearance via alveolar macrophages. As a result, a drug delivery system that combines the benefits of both nanocarriers and microparticles was created. Nanocomposites are made of NPs held together by a carrier, whereas nanoaggregates are made of NPs held together by van der Waals forces [168].

#### 4.3.1. Nanocomposites

Nanocomposites are essentially nanoparticles in microparticles. They are made up of drug-loaded nanoparticles and excipients (mostly sugars), which increase the size of the NPs to the micro-range (1–5 μm), making them suitable for deep lung deposition. The sugar then dissolves within the alveolar fluids, allowing the nanocarrier drug formulation to be released [169].

#### 4.3.2. Nanoaggregates

Nanoparticles can also be spray-dried into large hollow spherical nanoaggregates. These can then disassociate back into primary nanoparticles in the lung fluid. Nanoaggregates are of the order > 5 μm and hence can easily be agglomerated into inhalable formulations [170].

## 5. Toxicity Concerns

### 5.1. Toxicity Concerns Related to Inhalable Formulations

Nanocarriers may overcome drug solubility or stability issues and reduce drug-induced side effects. However, there may be considerable toxicity effects connected with the nanocarriers themselves. Upon inhalation of particulate drug carriers, either nano- or micro-sized, the lung shows a response in the form of increased macrophages. This is due to the deposition of non-biodegradable carriers in the lungs. At lower concentrations of the carrier, it is deemed as benign; however, continued exposure of these particles increases deposition being additive. This results in more severe inflammatory responses and could lead to a breakdown in the defense mechanisms of the lungs [171]. Particle size also plays an important role in determining where in the lung it gets accumulated. For nanoparticles of the order 1 nm, 90% can accumulate in the nasopharyngeal area, 10% accumulate in the tracheobronchial area, and almost nothing accumulates in the alveolar area. In contrast, nanoparticles of the order 20 nm, 15% accumulate in the nasopharyngeal area, 15% in the tracheobronchial area, and 50% in the alveolar area [172].

Inhalable nanocarriers cause significant toxicity in the form of oxidative stress and functional disturbances caused due to inflammation. In vivo studies carried out by Bermudez et al. [173] and Elder et al. [174] showed that inflammation in lungs could be caused by inhalation, while Warheit et al. [175] showed that it could be induced by instillation exposures. These studies revealed the local conquest of leukocytes, increased numbers of inflammatory cells in bronchoalveolar lavage fluid, LDH liberation, and increased cytokine production [173,174,175].

Underlying respiratory diseases also affect inhalation and deposition pattern of the carriers in the lungs. Damaged lungs showed greater deposition of NPs as compared to healthy lungs triggering local inflammation and intensifying the underlying disease, such as asthma, in the patients [171].

Hence, there arises a need and growing importance of conducting extensive research on nanotoxicity to the lungs. This is required to identify the optimal characteristics of nanoparticles in order to make them less toxic and more biodegradable, identify their pulmonary and systemic distribution patterns as well as effects on major organs in the body.

### 5.2. Toxicity and Safety Concerns as per NIOSH Guidelines

The National Institute for Occupational Safety and Health (NIOSH) prepares and revises recommendations for limits of exposure to potentially hazardous chemicals or circumstances in the workplace under the authority of the Occupational Safety and Health Act of 1970. NIOSH also suggests preventative strategies targeted to limit or eliminate the adverse health impacts of certain risks. In making these recommendations, NIOSH considers all known and accessible scientific evidence related to the possible threat. It establishes and regularly revises recommended exposure limits (RELs) for hazardous chemicals or circumstances in the workplace under the authority of the Occupational Safety and Health Act of 1970 and the Federal Mine Safety and Health Act of 1977. NIOSH also suggests suitable preventative strategies to mitigate or eliminate the negative health and safety impacts of these risks. NIOSH reviews all known and accessible medical, biological, engineering, chemical, trade, and other information related to the hazard in order to create these recommendations. These recommendations are then made public and sent to OSHA and MSHA. The likelihood of hazardous medications causing adverse effects on healthcare personnel, as well as the severity of the harm, is determined by various factors, including:

- Drug potency and its toxicity;
- Route of exposure;
- Drug’s physical and chemical properties;
- Type of drug formulation (such as liquid, powder, capsule, or pre-filled syringe).

Cytotoxic medications used to treat cancer in patients might be harmful, regardless of their composition or formulation. These medications have unique handling instructions from the manufacturer that must be followed at all times. Some medications are also considered dangerous by NIOSH because they fulfill NIOSH criteria for hazardous pharmaceuticals. These include cancer-causing medications as well as pharmaceuticals that impair a worker’s organs such as the liver, brain, kidney, or other organs. Some of the dangerous medications used to treat patient illnesses/cancer might injure unprotected healthcare professionals on the job. To safeguard healthcare employees, it is advised that employers identify the dangers particular to their workplace and establish hazard control techniques to reduce such hazards. These may include the use of engineering controls, such as ventilated hoods and enclosures; the implementation of administrative controls, such as the establishment of safe handling policies, training, and routine training reviews for potentially exposed individuals; and the provision of personal protective equipment (PPE), such as chemotherapy gloves, gowns, and respirators/protective face masks, when necessary.

## 6. Devices Used for Pulmonary Delivery

**Figure 8:**
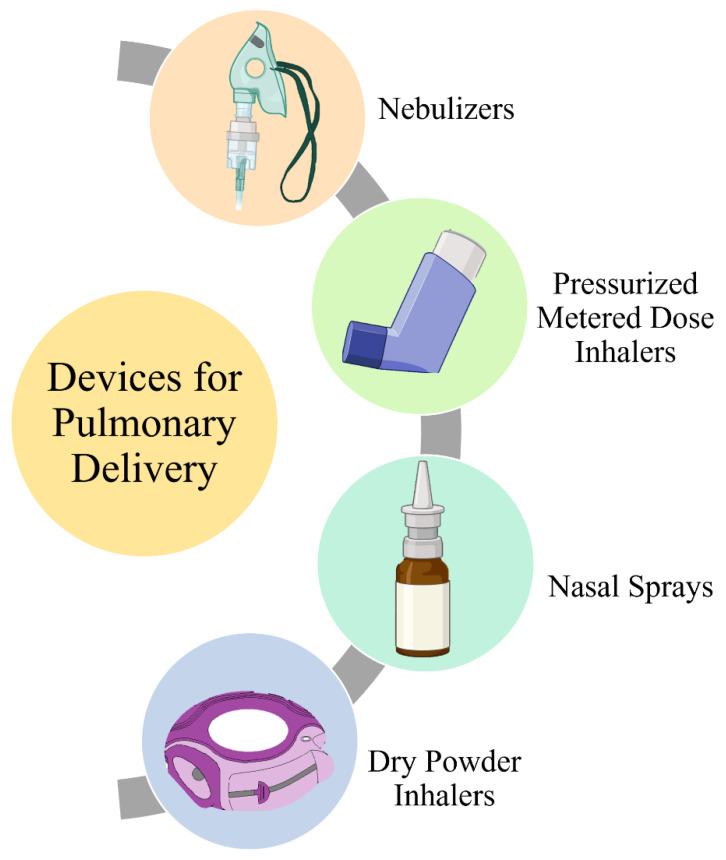
The different devices employed for pulmonary delivery of anticancer agents.

The formulations developed can be delivered using various devices. The main devices used commercially for intratracheal pulmonary administration are nebulizers, dry powder inhalers (DPIs), pressurized metered-dose inhalers (pMDIs), and nasal sprays (Figure 8).

Depending upon the dose, different devices can be used to administer drugs. pMDI and nasal sprays can usually deliver small (in the order of micrograms) doses only, which is not sufficient for an effective chemotherapeutic outcome [176]. However, doxorubicin-based dendrimers have been administered via pMDI in some studies [177]. Hence, DPIs and nebulizers are employed for delivering high doses of anticancer drugs intratracheally.

Nebulizers are used to deliver liquid-based aerosols to the lungs in the form of finely atomized droplets, and therefore only require simple formulations such as suspensions and solutions. The different types of nebulizers available are jet, vibrating mesh, and ultrasonic nebulizers [178]. They are the most widely used devices for clinical studies, especially air jet nebulizers [12,179]. They also pose the advantage of being easy to use, since no inhalation technique is required. They can also be used by patients who are unable to perform active inhalation (e.g., bedridden patients) or not capable of receiving mechanical ventilation [180]. However, nebulizers are not the most efficient way of administering drugs. Nebulization takes hours of administration and requires multiple cycles. It took up to 6 h, for example, to deliver cisplatin liposomes using an air jet nebulizer [181]. It is also thought to be ineffective in terms of lung deposition. This is because during nebulization, large amounts of the aerosolized drug is not inhaled and lost into the device or released into the air, contaminating both the device and the environment. Because of this, nebulized drugs must be administered in hospital settings to prevent contamination. Only about 10–15% of the drug is deposited into the lungs [182]. Gemcitabine, however, when delivered via a vibrating mesh nebulizer showed 43% lung deposition [183]. Water solubility is required for liquid formulations used for nebulization, which is difficult in most lipid-based formulations. Furthermore, the nebulization process also vastly affects the drugs. The size, drug loading capacity, and the in vitro release rate of these carriers is significantly impacted. Nebulizers use a liquid-based delivery system, which require a modification procedure to liquidize the lyophilized powder formulations. These liquid formulations present lower drug stability during long-term storage than a dry form. Hence, it is really important to consider the nebulization technique before aerosolization since the nanoparticle stability and eventually the therapeutic efficacy of the formulation will change [184,185].

Dry powder-based delivery systems are hence more beneficial than nebulization in particular for lung cancer treatment [186]. DPIs are simple to use, allow for self-administration, and do not require a hospital setting since they are portable, easily transportable, are less expensive, and can efficiently deliver high doses of anticancer drugs as dry powder to the lungs. Moreover, as compared to liquid formulations, DPI formulations are solid and therefore are more stable for long-term storage. They also do not need good water solubility and hence more suitable for conventional lipophilic anticancer drugs. DPIs can be manufactured as disposable devices, limiting device and environmental contamination [187,188,189,190]. The performance of DPIs is determined by the behavior of their composite particles. The cohesive and adhesive forces between the drug and carrier are crucial in determining the efficiency of the device. The drug might have high adhesive forces with the carrier, resulting in it getting stuck and not being released. As a result of the high adhesive and cohesive interactions, drug particles tend to stick to each other and to device surfaces, which is a disadvantage of DPIs [191,192].

**Figure 9:**
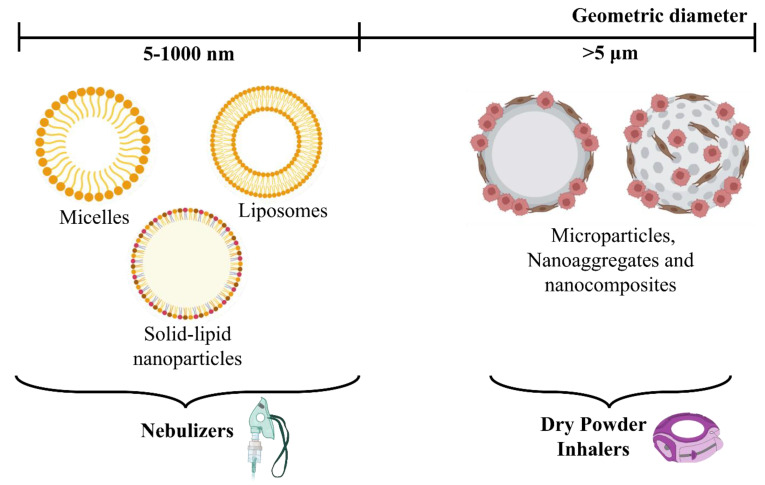
Drug delivery device choice based on the size of the formulation.

While developing formulations, the aerodynamic diameter is also an important factor to be considered. This is due to the fact that it is concerned with the dynamic behavior of a particle in an airflow, which is determined by its geometric size, shape, and density. Particles must have aerodynamic diameters of 1 to 5 μm to reach the lower respiratory tract and 1 to 3 μm to reach the respiratory zone (Figure 9) [193,194].

## 7. Conclusions

Conventional forms of therapy for lung cancer are now failing owing to their adverse side effects and ineffectiveness for treatment of later stage cancers. Inhalable therapy is a new and upcoming area of research. Inhalable anticancer treatment through different nanocarriers is an advanced and thrilling field that is nowadays being studied extensively. It is a promising treatment option for lung metastases and lung cancer. These carriers are attracting a lot of attention due to their distinctive properties of high drug loading, high biocompatibility, and tunable surfaces for active targeting and controlled drug-release behavior. The latter not only overcomes the drawbacks of conventional therapy, but also offers various advantages such as being non-invasive, biocompatible, and having improved pharmacokinetic factors. Due to being a targeted form of therapy, healthy tissues are not exposed to high concentrations of the drugs, resulting in decreased cytotoxicity. Nanocarriers have a high lung residence time due to their ability to avoid lung clearance mechanisms owing to their size. Microparticles on the other hand can aggregate into particles and be delivered via dry powder inhalers. Nanocomposites and nanoaggregates combine the advantages of both nanocarriers and microparticles into one formulation. Of all the devices available for pulmonary delivery of drugs, nasal sprays and pMDIs, cannot be used to administer high doses, making only nebulizers and DPIs suitable. DPIs have an advantage over nebulizers. This is because the former is cheap, can be self-administered, and does not contaminate the air as compared to the latter. The physicochemical properties of drugs play an important role in their formulations and the type of device that can be used for administration. At the in vivo and clinical study levels, this area of research is still in its infancy. Technique planning ought to focus on creating functional nanocarriers for dynamic focusing, with great medication stacking and supported drug release properties, implanted in all-round designed microparticles or nanocarriers made out of protected and very much endured excipients of high FPF for productive lung testimony, drug conveyance, and antitumor action. Despite all the advances, inhalable therapy is still relatively new and requires a lot of research before being applied in the clinical treatment of lung cancer.

## Figures and Tables

**Table 1:** Formulation strategies to overcome removal of drugs via lung clearance mechanisms.

Lung Clearance Mechanism	Strategy	Reference
Mucociliary Clearance	D_ae_ = 1.8–2.8 μm Increase mucus penetration with PEG-based formulation	[20,21,22]
Alveolar Phagocytosis	D_ae_ = 1–1.5 μm Vary particle size: nanoparticles, microparticlesVary geometric shape—worm-like-shaped particles	[23]
Dissolution	Micro and nanoparticles (lipid-, polymer-based) Liposomes	[22,24]

## Data Availability

Not applicable.

## Abbreviations

NSCLC	Non-small cell lung cancer
SCLC	Small-cell lung carcinoma
DPIs	Dry-powder inhalers
D_ae_	Aerodynamic diameter
EPR	Enhanced permeability and retention
PLGA	Polylactic-co-glycolic
PEG	Polyethylene glycol
CD-RES NP	Resveratrol-cyclodextrin nanoparticles
SF	Sorafenib
PLA	Poly-L-arginine
DOX	Doxorubicin
AQ	Amodiaquine
HPH	High-pressure homogenization
BSA	Bovine serum albumin
PEI	Polyethyleneimine
Cs	Chitosan
F	Fucoidan
HTCC	N-[(2-hydroxy-3- trimethylammonium)propyl] chitosan chloride
SLNs	Solid lipid nanoparticles
LPN	Lipid–polymer hybrid nanoparticles
NLCs	Nanostructured lipid carriers
SD	Sprague–Dawley
PFD	Pirfenidone
PTX	Paclitaxel
DLPC	Dilauroylphosphatidyl choline
CLH	Hydroxycamptothecin
5-ALA	5-aminolevulinic acid
5-FU	5-Fluorouracil
DPPC	Dipalmitoyl phosphatidylcholine
DPPG	Dipalmitoyl phosphatidylglycerol
DPPA	Dipalmitoyl phosphatidic acid
NEs	Nanoemulsions
PDI	Polydispersity index
LDH	Lactate dehydrogenase
CU	Curcumin
DOTAP	(N-[1-(2,3-dioleoyloxy)propyl]-N,N,N-trimethylammonium) or 1, 2-dioleoyl-3-trimethylammonium propane
LPHNs	Lipid–polymer hybrid nanoparticles
DLPC	Dilauroylphosphatidylcholine
9NC	9-Nitrocamptothecin
GPs	Gelatin nanoparticles
MNPs	Magnetic nanoparticles
MSNs	Mesoporous silica NPs
ZnO-NPs	Zinc oxide nanoparticles
Cp	Cisplatin
Sb-GNPs	Gemcitabine (Gem) silibinin-loaded gold nanoparticles
CMC	Critical micellar concentration
DTX	Docetaxel
pMDIs	Pressurized metered-dose inhalers
